# Anthrax Toxins Inhibit Neutrophil Signaling Pathways in Brain Endothelium and Contribute to the Pathogenesis of Meningitis

**DOI:** 10.1371/journal.pone.0002964

**Published:** 2008-08-13

**Authors:** Nina M. van Sorge, Celia M. Ebrahimi, Shauna M. McGillivray, Darin Quach, Mojgan Sabet, Donald G. Guiney, Kelly S. Doran

**Affiliations:** 1 Department of Biology and Center for Microbial Sciences, San Diego State University, San Diego, California, United States of America; 2 Department of Pediatrics, University of California San Diego, La Jolla, California, United States of America; 3 Department of Medicine, University of California San Diego, La Jolla, California, United States of America; The Research Institute for Children at Children's Hospital New Orleans, United States of America

## Abstract

**Background:**

Anthrax meningitis is the main neurological complication of systemic infection with *Bacillus anthracis* approaching 100% mortality. The presence of bacilli in brain autopsies indicates that vegetative bacteria are able to breach the blood-brain barrier (BBB). The BBB represents not only a physical barrier but has been shown to play an active role in initiating a specific innate immune response that recruits neutrophils to the site of infection. Currently, the basic pathogenic mechanisms by which *B. anthracis* penetrates the BBB and causes anthrax meningitis are poorly understood.

**Methodology/Principal Findings:**

Using an *in vitro* BBB model, we show for the first time that *B. anthracis* efficiently invades human brain microvascular endothelial cells (hBMEC), the single cell layer that comprises the BBB. Furthermore, transcriptional profiling of hBMEC during infection with *B. anthracis* revealed downregulation of 270 (87%) genes, specifically key neutrophil chemoattractants IL-8, CXCL1 (Groα) and CXCL2 (Groβ), thereby strongly contrasting hBMEC responses observed with other meningeal pathogens. Further studies using specific anthrax toxin-mutants, quantitative RT-PCR, ELISA and *in vivo* assays indicated that anthrax toxins actively suppress chemokine production and neutrophil recruitment during infection, allowing unrestricted proliferation and dissemination of the bacteria. Finally, mice challenged with *B. anthracis* Sterne, but not the toxin-deficient strain, developed meningitis.

**Conclusions/Significance:**

These results suggest a significant role for anthrax toxins in thwarting the BBB innate defense response promoting penetration of bacteria into the central nervous system. Furthermore, establishment of a mouse model for anthrax meningitis will aid in our understanding of disease pathogenesis and development of more effective treatment strategies.

## Introduction

*Bacillus anthracis* is a Gram-positive spore-forming bacterium that causes anthrax in humans and animals [1]. The recent threat of *B. anthracis* as a potential bioterrorism agent has sparked renewed interest into disease pathogenesis and treatment strategies. Infection occurs upon entry of bacterial spores through the skin, gastrointestinal mucosa or the lung [2]. Spores, initially taken up by resident macrophages [3] and dendritic cells [4], germinate to vegetative bacteria during phagocyte migration to the regional lymph nodes. Vegetative bacteria are then released from the phagocytes, enter the bloodstream [2] and proliferate in long chains at preferred sites like the brain, allowing entry into the central nervous system (CNS) and development of anthrax meningitis. The incidence of anthrax meningitis after cutaneous infection is approximately 5% [5], however in an outbreak of inhalational anthrax, approximately 50% of patients displayed signs of hemorrhagic meningitis [6]. Additionally, experimental studies of inhalational anthrax in monkeys demonstrated meningitis in 77% of cases examined [7]. In general, anthrax meningitis is associated with a fulminant and rapidly progressive deteriorating course approaching 100% mortality despite intensive antibiotic therapy [5].

The major virulence factors of *B. anthracis* are encoded on two native plasmids, pXO1 and pXO2 [2]. The pXO1 plasmid contains the toxin-gene complex comprised of protective antigen (PA), lethal factor (LF) and edema factor (EF) [1], [2]. These three toxin components combine to form two binary toxins, lethal toxin (LT), a zinc metalloprotease that cleaves mitogen activated protein kinases [8], and edema toxin (ET), an adenylate cyclase that increases intracellular cyclic AMP concentrations [9]. The pXO2 plasmid encodes genes involved in the production of the polyglutamyl capsule [1], [2]. Fully virulent strains of *B. anthracis* contain both plasmids, whereas the unencapsulated Sterne strain (pXO1^+^, pXO2^−^) is used for vaccination purposes [10]. In addition, the Sterne strain has been widely used in both *in vitro* and *in vivo* studies of anthrax infection as it causes lethal disease similar to the encapsulated *B. anthracis* strain in mice [11]. Currently however, no small animal model of anthrax meningitis exists that could facilitate our understanding of disease pathogenesis and the contribution of specific virulence factors to penetration of the CNS.

Several studies have demonstrated the presence of numerous Gram-positive bacilli in the cerebrospinal fluid and brain [5], [6], suggesting that *B. anthracis* is capable of breaching the blood-brain barrier (BBB). The human BBB, which is comprised principally of a single layer of specialized brain microvascular endothelial cells (BMEC), serves as a critical barrier to protect the CNS against microbial invasion. In addition to providing barrier function, the BBB has also been shown to play an active role in initiating a specific innate immune response promoting neutrophil recruitment [12], the clinical and diagnostic hallmark of acute bacterial meningitis. This response is thought to be effective in clearing bacteria from the cerebral microvasculature in the majority of BBB encounters with bacteria. We hypothesize that penetration of the BBB by *B. anthracis* likely involves bacterial invasion and transcytosis across brain endothelium, direct damage by bacterial toxins and/or activation of host inflammatory pathways that compromise BBB integrity. A comprehensive study of the BBB response to *B. anthracis* infection could therefore aid in our understanding of disease pathogenesis.

In this study, we examine for the first time the interaction of *B. anthracis* with the human BBB using a well established hBMEC model [13], specific pXO1 and isogenic toxin mutants and a newly-developed mouse model for anthrax meningitis. Our study demonstrates that *B. anthracis* penetrates brain endothelium directly and that anthrax toxins contribute to this process. Additionally, anthrax toxins suppress the BBB neutrophil recruitment response promoting unchecked bacterial replication within the CNS and establishment of meningitis in a newly developed model of hematogeneous anthrax meningitis.

## Results

### B. anthracis invades brain microvascular endothelial cells

**Figure 1:**
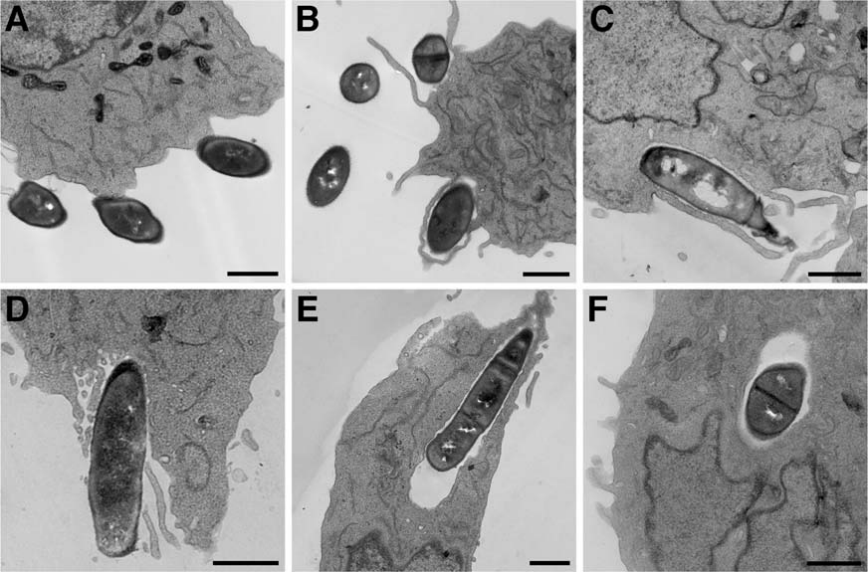
Transmission electron microscopy (EM) of hBMEC cells after infection with vegetative *B. anthracis* Sterne. (A, B) Transmission EM showing adherent *B. anthracis* Sterne to the cell membrane or in close proximity to microvillus projections of hBMEC. (C, D) Vegetative *B. anthracis* Sterne in the process of invading hBMEC or (E, F) inside membrane bound vesicles. Scale bar: 1 µm

Analysis of cerebral spinal fluid and brains from patients with anthrax meningitis show the presence of numerous Gram-positive bacilli [5], [6], indicating that *B. anthracis* is able breach the BBB. We hypothesized that *B. anthracis*, like other meningeal pathogens [14], [15], is able to invade human BMEC (hBMEC), a single-cell layer that comprises the BBB. We therefore examined *B. anthracis* Sterne interactions with hBMEC using transmission electron microscopy (TEM). After a 1 hour infection period, bacteria were observed in close association with the cell membrane (**Fig. 1A**) and in close proximity to cell surface microvillus projections (**Fig. 1B**). After further exposure numerous bacteria were found either entering hBMEC or in membrane-bound intracellular vacuoles (**Fig. 1C–F**).

**Figure 2:**
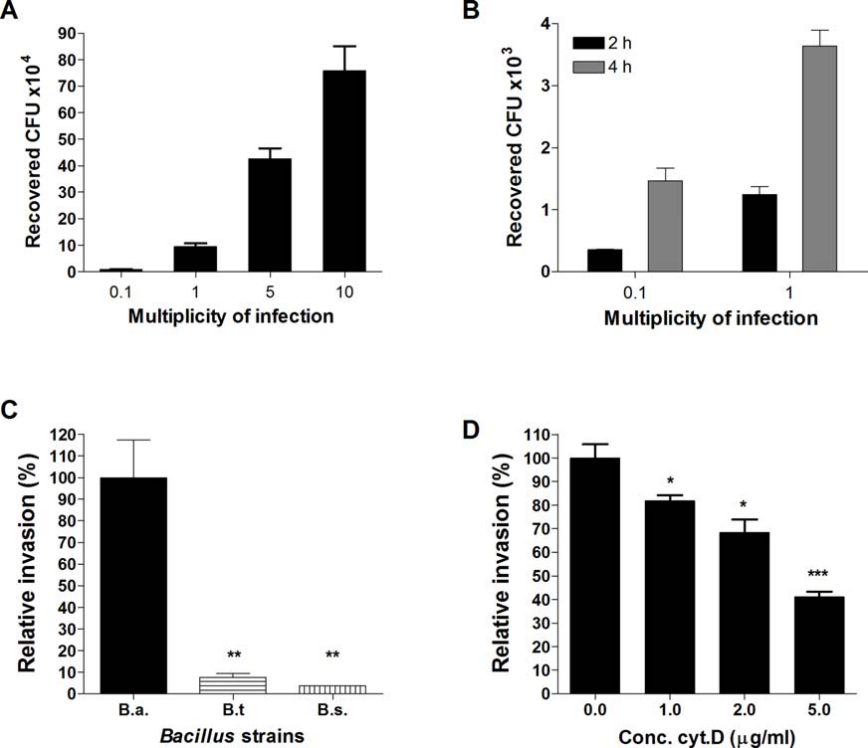
Interaction of *B. anthracis* Sterne with hBMEC. *B. anthracis* Sterne (A) adheres to and (B) invades hBMEC. Data are expressed as the recovered total cell-associated or intracellular colony forming units (CFU). MOI of 0.1, 1, 5, 10 is approximately 1×10^4^, 1×10^5^, 5×10^5^, and 1×10^6^ CFU respectively (C) Invasion of hBMEC by *B. anthracis* Sterne (B.a.) and closely-related *B. thuringensis* (B.t.) and *B. subtilis* (B.s.). (D) Concentration-dependent inhibition of *B. anthracis* hBMEC invasion by cytochalasin D, a potent inhibitor of actin cytoskeleton rearrangements. All experiments were repeated at least three times in triplicate, data from a representative experiment are shown. The error bars indicate 95% confidence intervals of the mean of three wells.

To quantify the number of adherent and invasive organisms, we optimized our previously established quantitative hBMEC adherence and invasion assays [12], [16] for *B. anthracis*. HBMEC were grown to confluency and infected with increasing concentrations (multiplicities of infection, MOI) of *B. anthracis* Sterne (MOI 1 represents approximately 1×10^5^ CFU); data are expressed as the recovered total cell-associated or intracellular colony forming units (CFU). The number of adherent bacteria steadily increased with increasing MOI (**Fig. 2A**) and ranged from 25–40% of the initial inoculum. Correspondingly, more intracellular organisms were recovered after infection with a higher MOI and longer incubation time (**Fig. 2B**), representing between 2–10% of the initial inoculum.

As the brain endothelium cells are responsible for maintaining biochemical homeostasis within the central nervous system (CNS), entry of molecules into the CNS is a strictly regulated process [17], [18]. However, to further demonstrate that the interaction of *B. anthracis* Sterne is not just due to random uptake, we incubated hBMEC with two related non-pathogenic Bacillus species, *B. thuringensis* and *B. subtilis*. Both of these strains were unable to invade hBMEC (**Fig. 2C**), demonstrating that the invasive ability is specific to *B. anthracis* Sterne.

The EM studies suggested that *B. anthracis* Sterne alters the host cytoskeleton to initiate its own uptake (**Fig. 1**). To confirm this observation experimentally, invasion experiments were performed in the presence of cytochalasin D, a potent inhibitor of cytoskeletal rearrangements. This inhibitor has been shown previously to effectively block invasion of hBMEC by other bacterial pathogens [16]. As shown in **Fig. 2D**, addition of cytochalasin D resulted in a dose-dependent inhibition of *B. anthracis* Sterne invasion into hBMEC. Together these results suggest that *B. anthracis* Sterne modulates the host cytoskeleton to induce it own uptake.

### Expression profile of hBMEC following B. anthracis Sterne infection

**Figure 3:**
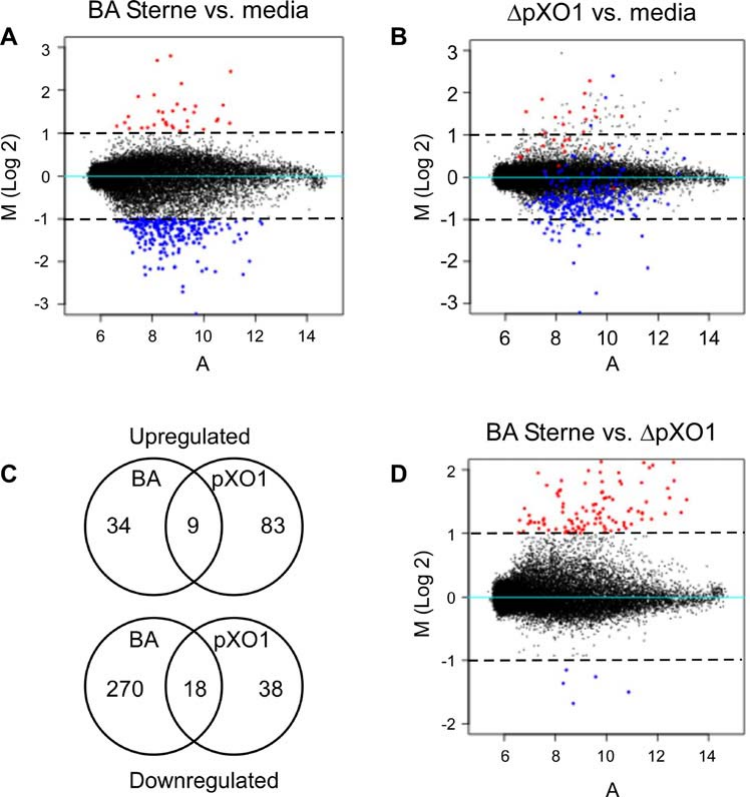
MA plots and Venn-diagram comparing the transcriptional response in hBMEC upon infection with *B. anthracis* Sterne or ΔpXO1 bacteria. MA plot showing the transcriptional profile of hBMEC upon infection with (A) *B. anthracis* Sterne vs. media control or (B) ΔpXO1 mutant bacteria vs. media control. Red and blue dots indicate genes that were more than two fold upregulated or downregulated, respectively upon infection with Sterne bacteria. These same genes retain their color in panel B. (C) Venn diagram depicting the number of up- or downregulated that were unique or overlapping in hBMEC upon infection with *B. anthracis* Sterne or ΔpXO1 mutant bacteria. (D) MA plot comparing gene expression of ΔpXO1/B.a.Sterne; red and blue dots indicate genes that were more than two fold differentially induced or suppressed, respectively.

Understanding changes in gene expression that occur in response to *B. anthracis* infection will facilitate further analysis of anthrax meningitis pathogenesis. We performed microarray analysis to assess the overall response of hBMEC to infection with *B. anthracis* Sterne. After 6 hours of infection, 304 genes exhibited a more than two-fold change in transcript abundance (**Table S1A,** **Fig 3C**). **Figure 3A** depicts the mean differences in total gene expression in Sterne-infected hBMEC cells compared to uninfected cells; down- and upregulated genes in response to *B. anthracis* Sterne are represented by blue and red dots, respectively. Interestingly, the majority of affected genes (270 out-of 304, 87%) were downregulated in response to *B. anthracis* Sterne infection compared to the uninfected control (**Fig 3A, C**). However, transcription was not globally impaired as 34 genes showed a more than two fold increased expression upon infection with *B. anthracis* Sterne (**Fig. 3A, C**, **Table S1A**).

### Differential gene expression profile induced by B. anthracis lacking the pXO1 plasmid

Anthrax toxins are considered to be the major virulence factors in *B. anthracis* infection. We therefore assessed the contribution of the pXO1 plasmid, encoding anthrax toxins, to the hBMEC transcriptional response. In parallel microarray studies, the *B. anthracis* Sterne ΔpXO1 strain affected 121 hBMEC genes by more than two-fold after 6 hours of infection compared to the uninfected control (**Fig. 3C**, **Table S1B**). In contrast to infection with the Sterne bacteria, only 38 out of 121 (31%) genes were downregulated upon infection with ΔpXO1 bacteria (**Fig. 3B, C**). The microarray data also show that mRNA levels for various housekeeping genes like β-actin and GAPDH were similar for samples infected with Sterne or the ΔpXO1 mutant (data not shown). To visualize how the down- and upregulated genes of Sterne-infected cells (colored blue and red, respectively, Fig. 3A) were affected in ΔpXO1-infected cells, the same genes were followed in the MA plot of ΔpXO1-infected hBMEC cells compared to media control (**Fig. 3B** **)**; 90% of genes were differentially affected in response to bacteria that lacked pXO1 (**Fig. 3B**). Also 40 additional genes were more than two fold induced in the absence of the pXO1 plasmid. Overall, this indicates that the hBMEC transcriptional response is strongly influenced by the presence of the pXO1 plasmid, whereas only 10% of the genes are regulated in a pXO1-independent manner (**Fig. 3A–C**).

**Figure 4:**
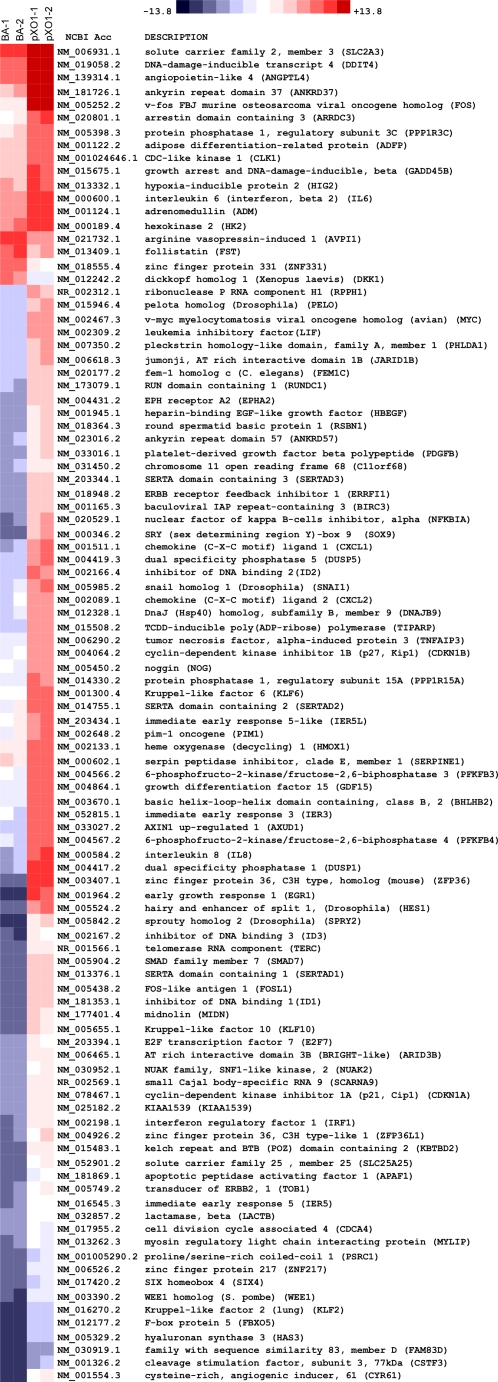
Heatmap identifying genes with 2.2 fold differential expression levels in hBMEC upon infection with *B. anthracis* Sterne vs. ΔpXO1 mutant strain. Each column represents a biological replicate microarray experiment upon infection with *B. anthracis* Sterne (BA) or ΔpXO1 bacteria (pXO1). Red and Blue coloring indicates induced or downregulated gene expression levels, respectively, of infected hBMEC vs. media control. Expression clustering was performed based on pairwise similarity, as described in Material and Methods.

Since gene expression was strongly influenced by the pXO1 plasmid, we next sought to identify the most differentially regulated genes in response to infection with *Bacillus* Sterne or ΔpXO1 mutant strains. **Figure 3D** displays mean differences in gene expression comparing ΔpXO1 versus *B. anthracis* Sterne infected hBMEC; more than two fold differentially down- and upregulated genes are colored blue and red, respectively. The identity of these genes and their expression in hBMEC compared to media are displayed in **Figure 4**. Analysis of differentially expressed genes by gene ontology (GO) of molecular function indicated significant overrepresentation of genes involved with transcription (*p* = 1.3×10^−12^), growth (*p* = 7.9×10^−6^), RNA binding (*p* = 1.7×10^−5^), protein kinase inhibition (*p* = 2.8×10^−5^), and cytokine activity (*p* = 6.7×10^−5^). Of particular interest however, was the observation that some of the most strongly differentially affected genes were potent neutrophil chemoattractants IL-8 (CXCL8), CXCL1 (Groα) and CXCL2 (Groβ), since we have previously shown that neutrophil recruitment is a major part of the innate host defense response against bacterial infection [12]. This was supported by GO analysis showing significant downregulation of genes with chemokine activity (*p* = 8.1×10^−4^) and correspondingly genes involved in induction of positive chemotaxis (*p* = 4.2×10^−5^) and leukocyte activation (*p* = 9.1×10^−4^). These microarray data suggest that factors on the pXO1 plasmid may interfere with the BBB innate immune defense, specifically neutrophil recruitment.

### Confirmation of microarray expression data: quantitative RT-PCR and ELISA

**Figure 5:**
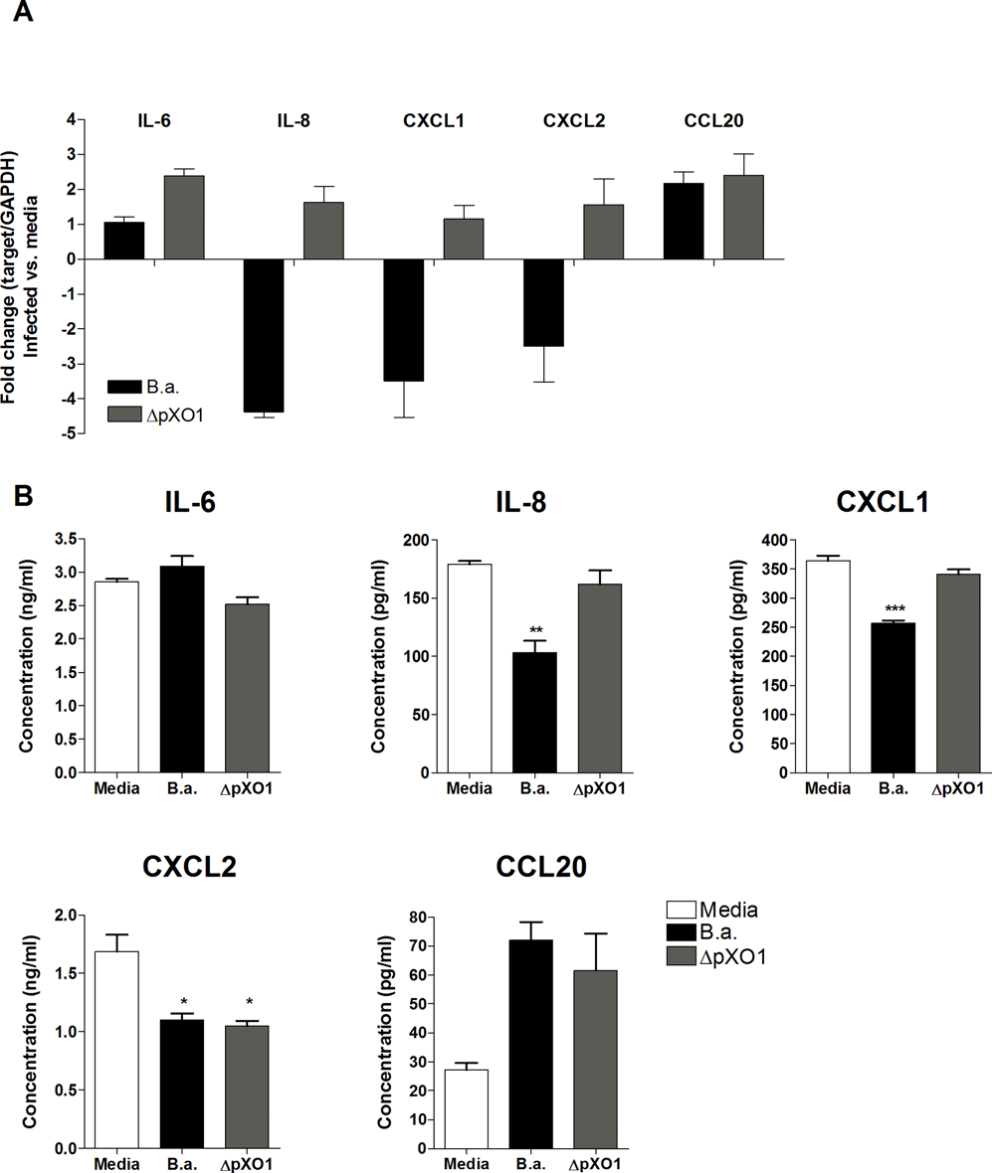
mRNA and protein expression of IL-6, IL-8, CXCL1, CXCL2 and CCL20 in hBMEC upon infection with *B. anthracis* Sterne (B.a.) or ΔpXO1 mutant bacteria. (A) mRNA expression levels of IL-6, IL-8, CXCL1, CXCL2 and CCL20 in hBMEC upon infection with *B. anthracis* Sterne (B.a.) or ΔpXO1 using quantitative RT-PCR. Fold change was determined as described in Material and Methods. Data represent mean and standard deviation of three independent experiments performed in triplicate. (B) Protein expression of IL-6, IL-8, CXCL1, CXCL2 and CCL20 in hBMEC supernatants 6 h post infection with *B. anthracis* Sterne (B.a.) or ΔpXO1 bacteria using ELISA. Experiments were performed three times in triplicate. Bars represent mean and standard deviation of one representative experiment. * *p*<0.05, ** *p*<0.005, *** *p*<0.001.

To confirm our microarray results we used quantitative RT-PCR to analyze the relative transcript abundance in hBMEC of the following genes involved in the host immune response: IL-6, IL-8, CXCL1, CXCL2, and CCL20. **Figure 5A** depicts the relative fold change in hBMEC transcript levels upon infection with *B. anthracis* Sterne or ΔpXO1 mutant bacteria compared to the uninfected control. As was observed in our microarray studies, the transcript levels of IL-8, CXCL1 and CXCL2 were significantly downregulated in cells infected with *B. anthracis* Sterne compared to uninfected control or hBMEC infected with the ΔpXO1 strain (**Fig. 5A**). In contrast, IL-6 and CCL20 transcripts were not downregulated in response to Sterne infection. This response was not significantly different upon infection with ΔpXO1 mutant bacteria, indicating that gene regulation for these genes is independent of the presence of the plasmid.

Effects on gene transcription are not always paralleled by changes in protein expression [19]. Therefore, we analyzed hBMEC supernatants for the presence of IL-6, IL-8, CXCL1, CXCL2, and CCL20 protein 6 hours after *B. anthracis* Sterne infection. Induction of chemokines IL-8, CXCL1 and CXCL2 was markedly reduced when cells were infected with the Sterne bacteria compared to uninfected controls (**Fig. 5B**). In contrast, IL-6 and CCL20 protein levels were unaffected and induced, respectively. Infection of hBMEC with the ΔpXO1 strain restored secretion of IL-8 and CXCL1 to levels secreted by uninfected cells, while IL-6, CXCL2, and CCL20 protein expression levels did not differ in the absence of the pXO1 plasmid compared to *B. anthracis* Sterne-infected hBMEC, suggesting that additional chromosomal factors may influence protein expression (**Fig. 5B**). Overall, these independent experiments generally confirmed our observations from the microarray experiment and suggest a role for pXO1-encoded factors in the downregulation of neutrophil chemokines in hBMEC.

### Anthrax toxins inhibit expression of IL-8 and suppress neutrophil recruitment in vivo

**Figure 6:**
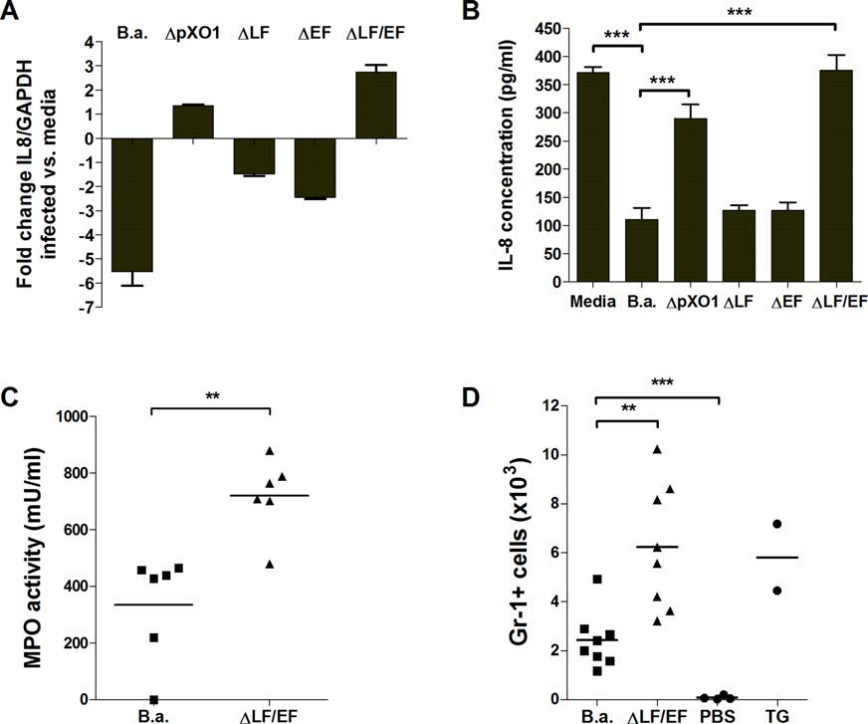
Anthrax toxins impair IL8 expression and neutrophil chemotaxis (A) IL-8 mRNA expression levels upon infection with *B. anthracis* Sterne (B.a.), ΔpXO1, ΔLF, ΔEF or ΔLF/EF mutant bacteria using quantitative RT-PCR. Data represent mean and standard deviation of three independent experiments performed in triplicate. (B) Protein expression of IL-8 in hBMEC supernatants 6 h post infection with *B. anthracis* Sterne, ΔpXO1, ΔLF, ΔEF or ΔLF/EF mutant bacteria using ELISA. Experiments were performed three times in triplicate. Bars represent mean and standard deviation of one representative experiment. Neutrophil recruitment *in vivo* was assessed by measuring (C) myeloperoxidase (MPO) activity in skin homogenates 4 h post infection or by (D) quantification of Gr-1+ cells upon peritoneal injection with *B. anthracis* Sterne (B.a.), toxin-deficient bacteria (ΔLF/EF), PBS or thioglycolate positive control (TG). Bars indicate mean levels of neutrophil recruitment. ** *p*<0.005; *** *p*<0.001.

To establish whether anthrax toxins were responsible for the downregulation of IL-8, the most potent and strongly affected neutrophil chemokine, we utilized isogenic mutants that specifically lacked LF (ΔLF), EF (ΔEF) or both anthrax toxins (ΔLF/EF). Infection of hBMEC with the ΔLF/EF bacterial strain resulted in a significant induction of IL-8 gene transcription (**Fig. 6A**) and restoration of IL-8 protein secretion (**Fig. 6B**) compared to *B. anthracis* Sterne infected cells. The presence of either LF or EF was still sufficient to suppress IL-8 transcript and protein expression (**Fig. 6A, B**), suggesting that both toxins are involved in downregulation of this neutrophil chemokine.

As the anthrax toxins decreased neutrophil chemokine transcription and expression, we hypothesized that neutrophil recruitment might be suppressed after infection with *B. anthracis* Sterne compared to the ΔLF/EF mutant strain. To examine the effects of anthrax toxins on neutrophil chemotaxis *in vivo*, we analyzed neutrophil recruitment to the site of infection using two independent assays. First, neutrophil recruitment was assessed upon subcutaneous injection of *B. anthracis* Sterne or the ΔLF/EF mutant strain into the right or left flank of mice, respectively, After 4 hours, mice were euthanized and the site of subcutaneous injection was excised, homogenized and analyzed for the neutrophil enzyme myeloperoxidase (MPO), which serves as an effective indicator of neutrophil infiltration [20]. MPO levels and therefore accumulating neutrophils were significantly lower upon infection with *B. anthracis* Sterne compared to the ΔLF/EF mutant strain (**Fig. 6C**). Using a second independent measurement, we quantified the amount of neutrophils entering the peritoneal cavity upon i.p. injection of *B. anthracis* Sterne or ΔLF/EF mutant bacteria. PBS and a 3% thioglycolate solution were included as negative and positive controls, respectively. After 4 hours, cells were extracted from the peritoneal cavity and the amount of accumulated neutrophils was quantified by flow cytometry. Although neutrophils were recruited upon infection by *B. anthracis* Sterne compared to the PBS control, neutrophil accumulation was significantly reduced compared to the toxin-deficient isogenic mutant (**Fig. 6D**). In general, neutrophil accumulation by the ΔLF/EF mutant was comparable to the positive 3% thioglycolate control (**Fig. 6D**). Overall, these results suggest that *B. anthracis* anthrax toxins interfere with transcription and secretion of neutrophil chemokines, as well as neutrophil recruitment during active infection.

### Anthrax meningitis mouse model

**Figure 7:**
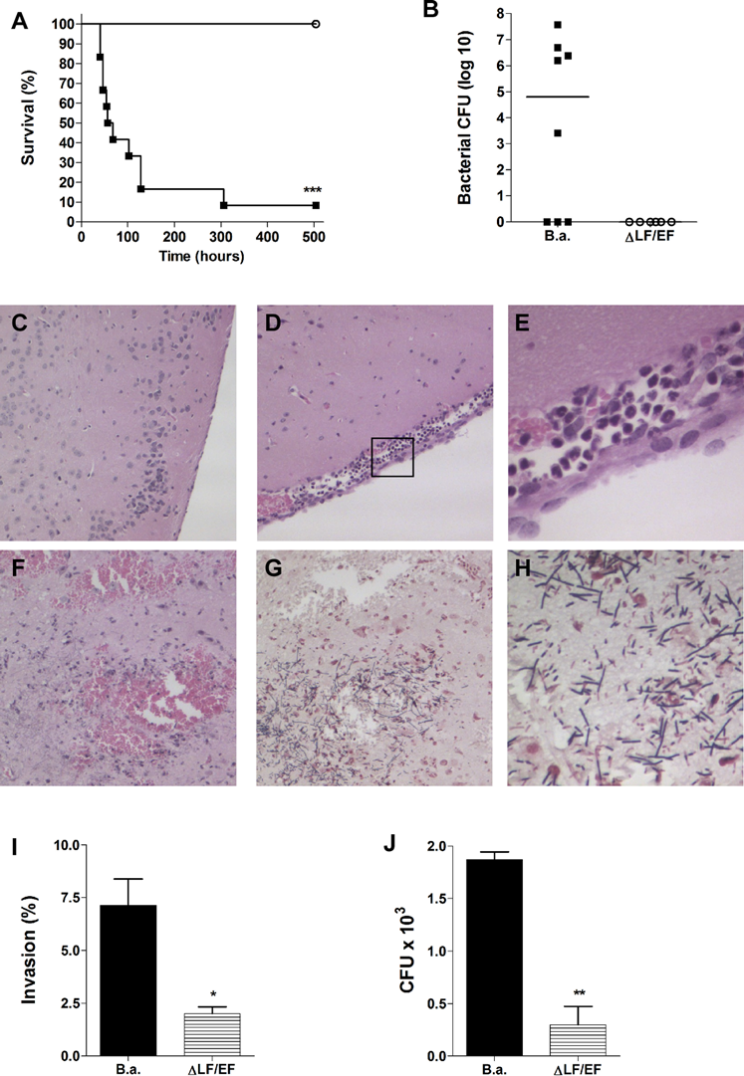
Mouse model of anthrax meningitis. (A) Kaplan-Meier survival curve of mice upon infection with *B. anthracis* Sterne (filled squares) or ΔLF/EF (open circles) bacteria. Groups of CD-1 mice (n = 12 per group) were injected intravenously with 2–3×10^4^ CFU of bacteria and survival was monitored at least twice a day over a three-week period. (B) Bacterial counts in brain at time of death of mice infected with *B. anthracis* Sterne (B.a.) or Δ LF/EF. Bar represents median bacterial number in the group of mice. Histopathology of (C–F) H&E- or (G, H) Gram-stained brain tissues of representative individual mice. (C) Sample from a mouse infected with ΔLF/EF bacteria showing normal brain architecture and no inflammation. Samples from mice infected with *B. anthracis* Sterne showing (D, E) meningeal thickening and cellular infiltration and (F) hemorrhaging. (G, H) Gram stain of a mouse infected with *B. anthracis* Sterne showing high levels of bacilli in the parenchyma. (I) Invasion and (J) transmigration of hBMEC by *B. anthracis* Sterne (B.a.) or ΔLF/EF bacteria. * *p*<0.05; ** *p*<0.005; *** *p*<0.001.

Our data suggest that *B. anthracis* is capable of penetrating the BBB. In addition, anthrax toxins suppress the brain endothelial host response which could promote unrestricted proliferation and further dissemination of *B. anthracis* in the CNS. To test the contribution of anthrax toxins to the pathogenesis of CNS infection, we developed a mouse model of anthrax meningitis. Mice were injected intravenously with *B. anthracis* Sterne or ΔLF/EF bacteria (n = 12 per group). Mice were euthanized when they became moribund with severely labored breathing (between days two and twelve for *B. anthracis* Sterne-infected mice) after which brain and blood were collected. All of the ΔLF/EF-infected mice and approximately 10% of the Sterne-infected mice survived until the experimental endpoint of three weeks (**Fig. 7A**). Five out-of eight Sterne-infected mice (63%) had high bacterial counts in the brain (**Fig. 7B**), while no bacteria were recovered from the brains of ΔLF/EF-infected mice (**Fig. 7B**). Microscopic examination of brain tissue from mice infected with the Sterne strain showed thickening of the meninges, an influx of inflammatory cells and substantial hemorrhaging (**Fig. 7D–F**). In addition, Gram stain revealed the presence of numerous bacilli in both the meninges and the parenchyma (**Fig. 7G, H**). The brains of mice that were infected with the ΔLF/EF strain did not show any signs of infection over the course of the experiment and exhibited normal brain architecture (**Fig. 7C**).

The absence of clinical symptoms in ΔLF/EF-infected mice could partially be due to reduced virulence of this strain *in vivo* [21]. Therefore, we performed additional *in vitro* experiments to assess whether anthrax toxins contribute directly to the penetration of brain endothelium. Compared to the parent strain, the toxin deficient strain exhibited a 70–80% reduction in hBMEC invasion (**Fig. 7I**) and was less able to penetrate hBMEC monolayers in a transmigration assay (**Fig. 7J**). Together these data indicate that *B. anthracis* Sterne is indeed capable of crossing the BBB *in vivo*, establishing the classic signs of meningitis and meningoencephalitis, and that the expression of anthrax toxins may directly contribute to this process.

## Discussion

Infection with *B. anthracis* resulting in systemic disease is associated with high morality characterized by septicemia, toxemia, and meningitis [6], [22]–[24]. The presence of bacilli in brain autopsies indicates that vegetative bacteria are able to disseminate from the bloodstream to the CNS, however, the basic pathogenic mechanisms by which *B. anthracis* penetrates the BBB have not been described. Using electron microscopy and an established *in vitro* model of the BBB, we demonstrate here for the first time that *B. anthracis* is capable of invading hBMEC, the single cell layer that comprises the BBB. Our observations extend recent studies reporting invasion of *B. anthracis* into non-phagocytic fibroblasts and epithelial cell lines [25]. Furthermore, our results suggest that uptake of *B. anthracis* Sterne in hBMEC is specific and requires actin cytoskeleton rearrangements. Interestingly, a very recent report identified a pXO1-encoded adhesin, BslA important for adherence to keratinocytes and lung epithelial cells [26]. Studies to identify and characterize additional factors involved in hBMEC adherence and invasion, including the BslA adhesin, are in progress.

We have used microarray analysis to examine the acute response of brain endothelium to infection with vegetative *B. anthracis* Sterne. We have shown previously that the BBB plays an active role in initiating a very specific innate immune response to bacterial infection by inducing gene expression of factors promoting neutrophil recruitment [12]. Most strikingly, *B. anthracis* infection reduced steady-state expression of 270 genes by more than two-fold corresponding to 87% of all affected gene transcripts. This contrasts typical host cellular responses to microbial pathogens where the number of host genes induced by infection is significantly higher than the number of down-regulated genes [12], [27]. The majority of downregulated genes were related to transcription, signal transduction, stress, host immune response, and proliferation. As anthrax toxins are the major secreted *B. anthracis* virulence factors, we also analyzed the gene expression profile of hBMEC upon infection with a strain lacking the pXO1 plasmid, ΔpXO1, which encodes both anthrax toxins. Ninety percent of affected genes upon *B. anthracis* Sterne infection were differentially affected upon infection with ΔpXO1 bacteria, and in total only 31% of genes in ΔpXO1-infected cells were downregulated. Additionally, approximately 10% of genes were regulated independently of pXO1, suggesting possible involvement of *B. anthracis* chromosomal factors to host response. Overall, these results suggest a major role for plasmid encoded factors and toxins in regulating the brain endothelial host response.

Of particular interest was the unambiguous effect on the expression levels of genes belonging to the CXC chemokine family, particularly the neutrophil chemotactic factors IL-8, CXCL1 and CXCL2 in response *B. anthracis* Sterne infection. Notably the expression levels of other major pro-inflammatory mediators such as TNFα and IL-1 were not affected by *B. anthracis* Sterne or ΔpXO1 infection. Neutrophil recruitment is thought to be part of the very first line of CNS defense against bacterial infection [12] as many Gram-positive and Gram-negative meningeal pathogens induce expression of these genes in hBMEC [12], [28], van Sorge et al. unpublished data). Active impairment of neutrophil recruitment could therefore benefit survival and proliferation of *B. anthracis*, as both spores and vegetative bacteria are efficiently killed by human neutrophils [29]. Our results clearly demonstrate that the suppression of CXCL1 and IL-8 expression is pXO1- and toxin-dependent, respectively. These data complement observations in recent studies where systemic infection with the encapsulated strain impaired production of cytokines in a toxin-dependent manner [30] and purified LT reduced IL-8 production by the destabilization of IL-8 mRNA in HUVEC *in vitro* [31].

We hypothesized that altered chemokine expression would result in impaired neutrophil recruitment upon active infection with *B. anthracis* Sterne. Using two independent *in vivo* assays, we demonstrated that neutrophil chemotaxis was indeed reduced to the site of infection with the Sterne strain as compared to infection with the ΔLF/EF mutant. Similar observations were recently published in a systemic infection model using encapsulated WT *B. anthracis* (pXO1^+^, pXO2^+^); host neutrophil recruitment in spleen and liver was significantly increased in the absence anthrax toxins compared to infection with the parent strain [32]. In addition, purified LT has been shown to directly impair neutrophil motility [33], [34]. Toxin-mediated subversion of the innate immune system, specifically targeting neutrophils, may therefore contribute to unchecked bacterial replication and a more fulminent disease course.

Establishment of an anthrax meningitis model is critical to better understand disease pathogenesis. The current rabbit and rhesus monkey models of inhalation anthrax [7], [35] both report signs of meningitis in a subgroup of animals, however, a mouse model would be preferable due to availability, lower costs and well-characterized genetic systems. We found that intravenous injection of immunocompetent outbred CD-1 mice with *B. anthracis* Sterne resulted in penetration of bacilli into the CNS. Microscopic analysis of brain sections confirmed the development of meningitis, showing inflammatory cell infiltration, hemorrhaging, thrombosis, edema and areas full of bacilli. While we did observe neutrophil infiltration in the brains of *B. anthracis* Sterne infected mice at the time of death, we speculate that an initial reduction or delay in host neutrophilic response may promote acute unrestricted bacterial proliferation and further CNS dissemination ultimately responsible for the rapidly progressive deteriorating course associated with anthrax meningitis. These observations reflect autopsy findings in patients [5] validating the utility of this newly developed mouse model of hematogenous anthrax meningitis. Finally, development of anthrax meningitis requires expression of anthrax toxins as no signs of disease developed in mice infected with the ΔLF/EF mutant strain. Additional *in vitro* studies suggested that this could be due to a direct contribution of the toxins to penetration of brain endothelium; however, we cannot exclude the possibility that the lack of clinical symptoms observed during infection with the toxin-deficient mutant may partially reflect a generalized reduction in virulence.

In summary, our studies provide the first evidence that *B. anthracis* is capable of invading the human BBB. We have also demonstrated that diverse functional classes of genes, including chemokines involved in neutrophil recruitment and signaling, were downregulated in brain endothelium upon *B. anthracis* infection suggesting that the pathogen actively suppresses the BBB innate immune response. This signaling appears to be mediated largely by the bacterial pXO1-encoded toxins. Our *in vivo* studies indicate that the anthrax toxins contribute to impaired neutrophil recruitment and the development of anthrax meningitis. Additional studies aimed at further understanding the mechanisms governing the pathogenesis of anthrax meningitis should aid in the development of preventative therapies for this serious CNS infection.

## Materials and Methods

### Bacterial strains and endothelial cell culture

*Bacillus anthracis* Sterne (pXO1^+^, pXO2^−^) and mutant derivatives were grown in Brain-Heart infusion broth (BHI; Sigma) as shaking cultures under aerobic conditions at 37°C. *B. anthracis* Sterne was cured of the pXO1 plasmid by passage at 43°C. Specific LF, EF and LF/EF deletion mutants were generously provided by Scott Stibitz (Center for Biologics Evaluation and Research, Bethesda, Maryland) and described previously [36]. For log-phase cultures of *B. anthracis*, fresh BHI was inoculated with the overnight culture at a 1∶20 dilution and grown to OD_600_ = 0.4 (1×10^7^ CFU/ml). Growth kinetics of all strains was similar under the experimental conditions used in our assays.

The human brain microvascular endothelial cell line hBMEC, obtained from Kwang Sik Kim (Johns Hopkins University, Baltimore, Maryland, USA), were originally isolated as previously described [13], [37], and maintain the morphologic and functional characteristics of primary brain endothelium [13], [15]. HBMEC were cultured using RPMI 1640 (Gibco), supplemented with 10% fetal calf serum (FBS; Gibco), 10% Nuserum (BD Biosciences, San Jose, California, USA), and modified Eagle's medium nonessential amino acids (Gibco) without addition of antibiotics. All experiments used cells at passage 8–14.

### HBMEC infection and transmigration assays

For hBMEC invasion assays, cells were seeded in collagen-coated 24 well tissue culture plates until they reached 90–100% confluency. *B. anthracis* cultures were grown to log-phase as described above. Log-phase bacteria were pelleted, washed in PBS and resuspended in RPMI 1640 10% FBS to the appropriate concentration. HBMEC monolayers, washed twice with PBS before the addition of bacterial cultures, were infected with different multiplicity of infection (MOI; MOI of 1 is approximately 1×10^5^ CFU) in a final volume of 500 µl of RPMI 10% FBS. Plates were centrifuged at 800× *g* for 5 min to synchronize the infection, and subsequently incubated at 37°C with 5% CO_2_. After 2–4 h, monolayers were washed three times with PBS before the addition of 1 ml of RPMI 10% FBS containing 50 µg of gentamicin for 15 min to kill extracellular bacteria. Control experiments confirmed that *B. anthracis* was killed by this concentration of gentamicin within 15 minutes (data not shown). The monolayers were washed three times with PBS before the addition of 0.1 ml of 0.25% trypsin/EDTA solution (5 min 37°C) followed by 0.4 ml of 0.025% Triton X-100 to liberate intracellular bacteria. The number of invasive bacteria was quantified by plating serial dilutions of the lysate on THB or BHI agar plates. To assess the effect of host cytoskeleton on *B. anthracis* invasion, hBMEC cells were incubated for 30 min with the indicated concentration of cytochalasin D (Sigma) before addition of bacteria. To assess the level of surface-adherent (total cell-associated) bacteria, bacteria were quantified from hBMEC monolayers prior to addition of extracellular antibiotics after 45 min of incubation as described above only washing six times with PBS prior to bacterial enumeration. All cellular adherence and invasion assays were performed at least in triplicate and repeated at least three times.

For transmigration assays, polar hBMEC monolayers were established on collagen-coated Transwell plates, 3 µm pore size (Transwell-COL; Corning-Costar Corp., MA, USA) as described previously [16]. Monolayers were incubated with 2×10^5^ CFU of log-phase grown bacteria. After 4 hours, the number of bacteria in the lower chamber was quantified by serial dilution plating on THA plates. The experiment was performed at least three times in triplicate.

### Transmission electron microscopy

Infection experiments were performed similar to the adherence assay described above with *B. anthracis* Sterne for 1 hour or 4 hours. After washing, samples were immersed in modified Karnovsky's fixative (1.5% glutaraldehyde, 3% paraformaldehyde and 5% sucrose in 0.1 M cacodylate buffer, pH 7.4) for at least 8 hours, post fixed in 1% osmium tetroxide in 0.1 M cacodylate buffer for 1 hour and stained en loc in 1% uranyl acetate for 1 hour. Samples were dehydrated in ethanol, embedded in epoxy resin, sectioned at 60 to 70 nm, and picked up on carbon-coated formvar grids. Grids were stained with uranyl acetate and lead nitrate, viewed using a JEOL 1200EX II (JEOL, Peabody, MA) or Philips CM-10 (FEI, Hilsboro, OR) transmission electron microscope and photographed using a Gatan digital camera (Gatan, Peabody, CA).

### Microarray analysis

Microarray experiments were performed using Sentrix Human-8 Expression BeadChips, which analyzed 25,440 transcripts (Illumina, San Diego, CA) according to manufacturer's instructions. In brief, a 250 ng aliquote of total RNA, isolated as described above, from each sample was amplified to cDNA, transcribed to cRNA and biotin labelled using Ambion's TotalPrep kit (Austin, TX), according to the instructions. cRNA concentrations were checked with the Agilent Bioanalyzer, and cRNA quality was controlled by BioRad's Experion Automated Electrophoresis System and RNA Std Sens Analysis Kit (BioRad Laboratories, Hercules, CA). Each sample cRNA (750 ng) was hybridized to Illumina's Sentrix Human-8 Expression BeadChip arrays at 58°C overnight (18 h, shaking) following the Illumina Whole-Genome Gene Expression Protocol for BeadStation. Hybridized biotinylated cRNA was detected with 1 mg/ml streptavidin-Cy3 (Amersham Biosciences, Piscataway, NJ). BeadChips were scanned with Illumina BeadArray Reader. Data was analyzed using a statistical algorithm developed for high-density oligonucleotide arrays [38].

### RNA isolation, cDNA preparation and qPCR

HBMEC monolayers were infected with *B. anthracis* Sterne, or isogenic mutants (ΔpXO1, ΔLF, ΔEF, or ΔLF/EF) for 6 hour. Total RNA was extracted using the RNeasy kit (Qiagen, Valencia, CA) according to the manufacturer's instruction, and 1 µg of RNA reverse transcribed to cDNA (Superscript First-strand synthesis kit, Invitrogen). Quantitative PCR (qPCR) was performed using the following primer sets: IL-6 forward primer 5′- GGA GAC TTG CCT GGT GAA AA -3′ and IL-6 reverse primer 5′- CAG GGG TGG TTA TTG CAT CT -3′, IL-8 forward primer 5′- AGC TCT GTG TGA AGG TGC AG - 3′ and IL-8 reverse primer 5′- AAT TTC TGT GTT GGC GCA GT - 3′, CXCL1 forward primer 5′ - CTC TTC CGC TCC TCT CAC AG - 3′, and CXCL1 reverse primer 5′ - GGG GAC TTC ACG TTC ACA CT -3, CXCL2 forward primer 5′- CTC AAG AAT GGG CAG AAA GC -3′, and CXCL2 reverse primer 5′- AAA CAC ATT AGG CGC AAT CC -3′, CCL20 forward primer 5′- GCG CAA ATC CAA AAC AGA CT -3′ and CCL20 reverse primer 5′- CAA GTC CAG TGA GGC ACA AA -3′, and GAPDH forward primer 5′- GAA GGT GAA GGT CGG AGT CAA CG -3′ and GAPDH reverse primer5′- TCC TGG AAG ATG GTG ATG GGA T -3′. PCR reaction mixtures contained primers at a concentration 10 µM and PCR mix (SYBR GreenER qPCR Supermix for iCycler, Invitrogen) in a volume of 25 µl. qPCR cycling was as follows for all genes: 50°C for 2 min, 95°C for 7 min, followed by 40 cycles of 95°C for 15″ and 61°C for 1 min. Melting curve analysis was performed according to the manufacturer's instructions; PCR primer efficiencies were as follows: 1.92 for IL-6, 1.8 for IL-8, 1.83 for CXCL1, 1.99 for CXCL2, 1.94 for CCL20 and 1.88 for GAPDH. Calculation of relative gene expression included adjustments for PCR efficiencies and using the following equation: Relative gene expression = target gene efficiency×(C_T_ control - C_T_ sample)/1.88×(C_T_ control - C_T_ sample).

### Chemokine secretion in hBMEC supernatants

HBMEC supernatants were collected after infection with *B. anthracis* Sterne, ΔpXO1, ΔLF, ΔEF, or ΔLF/EF deletion mutants after 6 hours. Concentrations of IL-8 (R&D systems, Minneapolis, MN, USA), CXCL1 (R&D systems), CXCL2 (BioSupplyUK) and CCL20 (R&D systems) were measured using enzyme-linked immunosorbent assays (ELISA) according to the manufacturer's instructions. IL-6 and IL-8 concentrations were measured using the cytometric bead array system according to the manufacturer's instructions (BD Biosciences, Human inflammation kit).

### Mouse infection studies

All animal experiments were approved by the Committee on the Use and Care of Animals, and performed using accepted veterinary standards. For the meningitis model, bacteria were grown to early log phase, washed in PBS and resuspended to an optical density of 0.4 in PBS. Vegetative bacteria were diluted in PBS to 2–3×10^5^ CFU/ml and 0.1 ml was injected intravenously into 8 weeks old out bred immunocompetent female CD-1 mice (Charles River Laboratories, Wilmington, MA, USA). Mice were monitored for signs of infection at least twice a day for up to three weeks and euthanized when they became moribund. Blood and brain were collected and plated to determine bacterial counts. Half of the brain was stored in 10% formalin for further histology analysis performed at the UCSD Histopathology Core Facility (N. Varki, Director).

To determine neutrophil recruitment *in vivo*, *B. anthracis* Sterne and ΔLF/EF mutant bacteria were grown to early log phase, washed and resuspended in PBS to and OD_600_ = 0.4. Eight week old CD-1 female mice were injected with 1×10^6^ CFU of *B. anthracis* Sterne on the right shaved flank and with 1×10^6^ CFU of ΔLF/EF mutant bacteria on the left shaved flank in a volume of 0.1 ml. After 4 hours, mice were euthanized and the site of subcutaneous injection was excised for further analysis of myeloperoxidase activity (see below). Neutrophil recruitment was also assessed using an intraperitoneal infection model. Eight week old CD-1 female mice were injected i.p. with 2×10^6^ CFU in 200 µl PBS. PBS alone and a 3% thioglycolate solution were used as negative and positive control for neutrophil recruitment, respectively. After 4 hours, cells were harvested from the peritoneal cavity in PBS 0.2% BSA. One-hundred µl of cell suspension was directly stained with FITC-labeled rat anti-mouse Gr-1 monoclonal antibody or the appropriate isotype control (both BD Pharmingen) for 30 min at 4°C and analyzed by flow cytometry. The flow cytometer was set to count events during a fixed time (60 s) thus permitting quantification of the absolute number of recovered Gr-1 positive cells in each mouse [39]. A quality check was performed on the flow cytometer (Dual Laser FACSCalibur Flow Cytometer) before use to assure a constant flow rate.

### Myeloperoxidase assay

Skin samples of mice were homogenized in 500 µl 0.05% hexadecyltrimethylammonium bromide (HTAB in 0.05 M phosphate buffer, pH 6; Sigma) solution. Homogenates were centrifuged for at 18,000× *g* for 30 min at 4°C. Supernatants were transferred to a clean microcentrifuge tube and stored at −80°C until further analysis. Next, 10 mg of *o-*dianisidine dihydrochloride (DCC; Sigma) was added to 60 ml of freshly-prepared HTAB solution to yield DCC solution. In addition, activated substrate was prepared by adding one µl of 0.05% hydrogen peroxide solution for every 99 µl of DCC solution. Finally, the reaction was started by adding 90 ul of DCC solution in HTAB solution and 100 ml of activated solution to 10 µl of skin supernatants 96 well flat-bottom plates. The absorbance was read every minute for 10 minutes at 450 nm using a spectrophotometer. All samples were analyzed in triplicate. For quantification purposes, a calibration curve of horseradish peroxidase (Calbiochem) ranging from 100 mU/ml to 3.13 mU/ml was run in parallel with the samples in triplicate with every experiment.

### Statistical analysis

Graphpad Prism version 4.03 was used for statistical analysis. Differences in adherence/invasion, mRNA expression, chemokine secretion in hBMEC supernatants were evaluated with a one-way ANOVA followed by Tukey's post hoc test. Differences in neutrophil recruitment were determined using a paired *t*-test for the MPO assay and an unpaired *t*-test for the intraperitoneal infection model. Kaplan-Meier survival plots were evaluated with the log-rank test. Statistical significance was accepted at *p*<0.05.

## Supporting Information

Table S1 A. Genes affected in hBMEC >2 fold by infection with B. anthracis Sterne B. Genes affected in hBMEC >2 fold by infection with pXO1 deficient mutant
(0.07 MB XLS)
